# Reconstruction of the Quadriceps Extensor Mechanism with a Calcaneal Tendon–Bone Allograft in a Dog with a Resorbed Tibial Tuberosity Fracture

**DOI:** 10.3390/ani14162315

**Published:** 2024-08-09

**Authors:** Hyunho Kim, Haebeom Lee, Daniel D. Lewis, Jaemin Jeong, Gyumin Kim, Youngjin Jeon

**Affiliations:** 1 Department of Veterinary Surgery, College of Veterinary Medicine, Chungnam National University, Daejeon 34134, Republic of Korea; wwkhww@hanmail.net (H.K.); seatiger76@cnu.ac.kr (H.L.); klmie800@cnu.ac.kr (J.J.); 2 Department of Small Animal Clinical Sciences, College of Veterinary Medicine, University of Florida, Gainesville, FL 32610, USA; lewisda@ufl.edu; 3 College of Veterinary Medicine, Jeonbuk National University, Iksan 54596, Republic of Korea; vetkgm@naver.com

**Keywords:** tibia tuberosity reconstruction, tendon injury, allogenic tendon transplantation, quadriceps mechanism restoration

## Abstract

**Simple Summary:**

This case report describes the surgical technique and clinical outcome of reconstructing the quadriceps mechanism using a composite frozen calcaneal tendon–bone block allograft in a dog which had resorption of the tibial tuberosity following complications resultant from a tibial tuberosity transposition procedure. In this case, a dog exhibited chronic lameness due to the resorption of the tibial tuberosity following surgical correction of a medial patella luxation. To address these issues, a novel surgical procedure was employed using a composite calcaneal tendon–bone block allograft. The graft reconstructed the tibial tuberosity and facilitated reattachment of the patellar tendon. The surgery promptly restored the quadriceps extensor mechanism, enabling the dog to bear weight on the affected limb within 2 weeks. Twenty-nine months later, the dog had satisfactory limb function without recurrence of patella luxation. This case demonstrates the effectiveness of using a calcaneal tendon–bone allograft to restore the quadriceps extensor mechanism in dogs with irreparable tibial tuberosity fracture.

**Abstract:**

A non-reducible tibial tuberosity fracture is a rare complication of tibial tuberosity transposition performed during correcting of medial patella luxation (MPL) in dogs. This condition severely disrupts the quadriceps extensor mechanism, leading to significant pelvic limb lameness. An 11-year-old, 1.8 kg spayed female Yorkshire Terrier sustained a comminuted left tibial tuberosity fracture during surgical correction of an MPL. Six months after surgery, the dog was markedly lame and unable to extend the left stifle. Radiographs revealed patella alta and resorption of the fragmented tibial tuberosity. A composite frozen allogeneic calcaneal tendon–bone block was utilized to reconstruct the tibial tuberosity and reattach the patellar ligament. Initial postoperative radiographs confirmed restoration of a normal patellar ligament to patella length ratio (1.42). Both the allogeneic bone used for tibial tuberosity reconstruction and the tendon used to reattach the patellar ligament were successfully integrated. The dog regained satisfactory limb function without recurrence of patella luxation, as reported by the owners 29 months postoperatively. The use of a calcaneal tendon–bone allograft effectively restored the functional integrity of the quadriceps extensor mechanism, providing a viable option for addressing quadriceps insufficiency resulting from the loss of the osseous tibial insertion.

## 1. Introduction

Unrepairable tibial tuberosity fractures are a rare but significant complication following tibial tuberosity transposition performed to address medial patella luxation (MPL) in affected dogs [1,2]. Fractures of the tuberosity can occur if the tuberosity segment is too small: either while performing the osteotomy or during the placement of Kirschner wires intended to stabilize the transposed tuberosity [1,2]. Fragmentation of the tibial tuberosity can severely impair the quadriceps extensor mechanism and limb function. Restoration of quadriceps function can be challenging as the remaining bone fragments attached to the patellar ligament may be too small to accommodate adequate fixation. Consequently, non-reducible tibial tuberosity fractures may necessitate a salvage procedure, such as stifle arthrodesis or amputation [3].

The calcaneal tendon has high tensile strength and a long, broad aponeuroses, making calcaneal tendon allografts a suitable structural and functional replacement option for ligament reconstruction in human patients [4,5,6]. The allogenic tendon acts as a scaffold to facilitate cellular migration and functional ingrowth, which eventually leads to parenchymal remodeling and integration with the native tissues, yielding functionality [4,6]. An attached segment of calcaneal bone is often harvested with the tendon to enable osseous fixation of the graft [7,8]. These attributes have contributed to the effective use of calcaneal tendon–bone allografts in human patients for various ligament reconstructions, including addressing anterior cruciate ligament, calcaneal tendon, medial collateral ligament, and biceps tendon insufficiencies [9,10,11]. Several studies have described the effective use of calcaneal tendon–bone allografts to manage quadriceps extensor mechanism insufficiency in human patients with patellar tendon rupture [12,13,14]. While there are a couple of reports detailing the use of calcaneal tendon allografts to augment tendon-to-tendon repairs in dogs with patellar tendon injuries [15,16], the application of a calcaneal tendon–bone allograft to address irreparable tibial tuberosity fractures has not been reported. 

This case report describes the surgical technique and clinical outcome associated with the use of a composite calcaneal tendon–bone block allograft to restore the quadriceps extensor mechanism in a dog with chronic lameness ascribed to an unrepairable tibial tuberosity fracture. Implantation of a calcaneal tendon–bone block allograft may be a reasonable option to address non-reducible tibial tuberosity fractures.

## 2. Case Description

An 11-year-old spayed female Yorkshire Terrier weighing 1.8 kg was presented with a history of chronic left pelvic limb lameness. The dog had undergone surgical correction of a grade III/IV MPL 6 months previously. During that surgery, the tibial tuberosity fragmented while attempting to perform a tibial tuberosity transposition. Fragmentation was sufficient to preclude reattachment of the patella ligament to the proximal tibia. An attempt was made to suture the distal portion of the patella ligament to the tibial crest using an absorbable 2-0 polydioxanone suture (PDS plus, Ethicon, Raritan, NJ, USA). Following surgery, the dog was unable to effectively extend the affected stifle or support weight on the limb.

**Figure 1:**
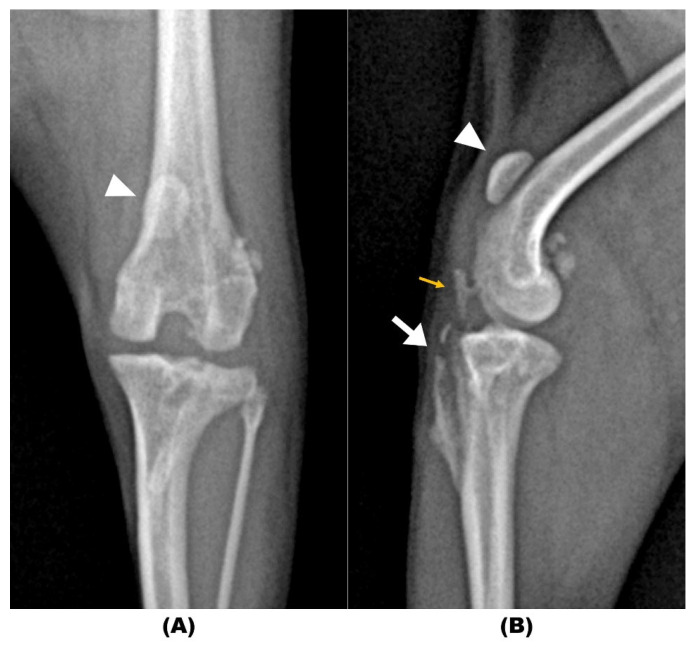
(**A**) Craniocaudal and (**B**) mediolateral radiographic views of the left stifle taken six months following initial surgery to address a grade III/IV medial patellar luxation. The images display tibial tuberosity absorption (white arrow) and proximal dislocation of the patella (arrowhead). Additionally, an ossicle (approximately 5 mm × 1.5 mm) with reduced bone density cranial to the infrapatellar fat pad was observed (yellow arrow).

On physical examination, the dog could not support weight on the left pelvic limb and maintained the limb in a flexed posture. The thigh girth of the affected limb was decreased by 20% compared to the contralateral pelvic limb. A patellar reflex could not be elicited in the left pelvic limb. Cranial drawer and cranial tibial thrust were negative in both stifles. Radiographs of the left stifle revealed proximomedial displacement of the patella relative to the femoral trochlear groove, consistent with patella alta and the absence of the normal protuberance of the tibial tuberosity (Figure 1). 

**Figure 2:**
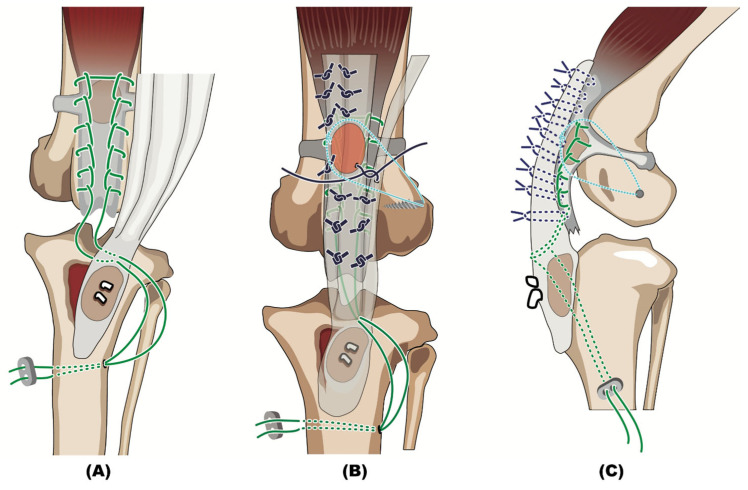
Schematic diagrams illustrating the surgical technique. (**A**) The osseous segment of the calcaneal tendon–bone allograft is secured within the prepared tibial bone bed using two Kirschner wires. A non-absorbable suture is placed through the proximal tibia, patellar ligament, and proximal patellar tendon. (**B**) Craniocaudal and (**C**) mediolateral images show that the allogenic tendon is augmented to enhance both the patellar ligament and the quadriceps tendon. Additionally, an encircling patellar suture is placed as a supplemental restraint to medial luxation.

Surgery involving implantation of a calcaneal tendon–bone allograft to restore the quadriceps extensor mechanism was planned (Figure 2). Tissues had been previously aseptically harvested, with the owner’s consent, from a 3-year-old, 4 kg, spayed female mongrel dog euthanized after sustaining a cervical vertebral fracture. The calcaneal tendon included the tendons of the superficial digital flexor, gastrocnemius, and the conjoined tendons from the gracilis, semitendinosus, and biceps femoris muscles and an attached 5 mm × 12 mm bone block were harvested from the calcaneal tuberosity. The tissues were packaged in a sterile container with an antibiotic solution consisting of gentamicin (200 μg/mL), vancomycin (100 μg/mL), and meropenem (200 μg/mL) [17]. The sterile container was sealed within an additional sterile plastic enclosure and frozen at −70 °C. The time from harvesting until use was 4 months. In preparation for implantation, the allograft was thawed in 37 °C sterile physiological saline for 30 min and small pieces of the tendon and bone were excised and submitted for bacterial culture to document that the graft had not been contaminated during the storage period. 

**Figure 3:**
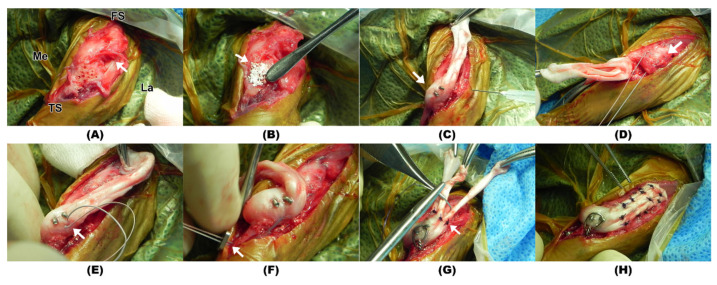
Intraoperative gross photographs. (**A**) Debrided bone displaying multiple holes for osteostixis. A noticeable gap is observed between the distal patellar ligament and the tibial bed (arrow). (**B**) The recombinant human bone morphogenetic protein-2 with hydroxyapatite applied to the bone surface (arrow). (**C**) Allogenic calcaneal bone block secured to the tibial bed using Kirschner wires (arrow). (**D**) A Krackow suture (arrow) initiated at the medial margin of the patellar ligament, encircling the proximal patella, and terminating at the lateral margin. (**E**) Two free ends of the suture (arrow) exit at the calcaneal bone–tendon allograft junction. (**F**) Both ends of the suture are threaded through the tibia and anchored with a suture button (arrow). (**G**) The grafted tendon is affixed to the patellar ligament and quadriceps tendon using multiple simple interrupted sutures (arrow). (**H**) Gross morphology of the completed implantation of the allograft. FS: femur side; TS: tibia side; Me: medial; La: lateral.

The dog was premedicated with hydromorphone (0.1 mg/kg intravenously [IV]) and midazolam (0.2 mg/kg IV). Anesthesia was induced with propofol (4 mg/kg IV) and maintained with 1.5% isoflurane in oxygen. Cefazolin sodium (22 mg/kg IV) was administered 30 min before skin incision and every 90 min during surgery. Intraoperative analgesia was provided using remifentanil (0.1–0.3 µg/kg/min constant rate infusion). A lateral parapatellar approach to the left stifle and proximal tibia was performed [18]. Fibrosis extended cranioproximal from the tibial crest to the distal stump of patellar ligament. A single 5 mm × 1.5 mm bone fragment was visible on the preoperative radiographs and presumed to be a remnant of the tibial tuberosity, but it could not be identified during surgery. After debridement of adherent fibrous tissue, there was an approximately 15 mm gap between the distal end of the patella ligament and the proximal extent of the tibial crest when the stifle was placed in an extended position (Figure 3A). The end of the distal patellar ligament could not be sufficiently mobilized to reattach to the tibia. A lateral parapatellar arthrotomy and medial retinacular release were performed. The cruciate ligaments were intact, and the trochlear groove had sufficient depth to maintain reduction of the patella. The cranioproximal surface of the tibial crest was debrided using a pneumatic burr to remove the granulation tissue which was adhered to the bone. A 0.7 mm Kirschner wire was used to create several holes in the debrided bone bed to expose bleeding cancellous bone. Recombinant human bone morphogenetic protein-2 (rhBMP-2) with hydroxyapatite (HA) (NOVOSIS-Dent, CGBio, Seongnam, Republic of Korea) was placed on the debrided osseous surface (Figure 3B). A mixture of 0.25 mg rhBMP-2 and 0.25 mg of HA was used.

The allograft bone block was secured along the lateral margin of the debrided tibial bone bed using both a 1.1 mm and a 0.9 mm interfragmentary Kirschner wire (Figure 3C). Lateralization of the allogenic bone block established appropriate alignment of the quadriceps mechanism. A strand of multifilament 0 braided non-absorbable suture (FiberWire, Arthrex, Naples, FL, USA) was placed through the proximal tibia, patellar ligament, and proximal patellar tendon to neutralize the force of the quadriceps muscles. Specifically, a Krackow suture pattern was initiated at the distal end of the medial margin of the patellar ligament and continued proximally and passed hemi-circumferentially around the proximal pole of the patella, then coursing distally, with the strand of suture exiting at the distal end of the lateral margin of the patellar ligament (Figure 3D). The two free ends of the suture, emerging from the medial and lateral margins of the distal patellar ligament, passed from the interior to the exterior of the graft tendon at the calcaneal bone–tendon allograft junction (Figure 3E). A transverse bone tunnel was subsequently created through the crainoproximal aspect of the proximal tibia using a 1.4 mm Kirschner wire, and the two free ends of the suture strands were threaded through this tunnel. The suture strands were tensioned to align the patella normally within the trochlear groove, with the stifle positioned at 135° of extension, and the strands were secured through a suture button, and a series of square knots were tied (Figure 3F). A strand of 21-gauge orthopedic wire was placed in a figure-of-eight pattern around the protruding ends of the two interfragmentary Kirschner wires and a transverse hole drilled through the tibia subjacent to the tibial crest, to prevent avulsion of the allograft bone block (Figure 3G). The lateral parapatellar arthrotomy was closed using simple interrupted sutures with 4-0 PDS. To further mitigate the potential of the patella to luxation medially, a suture anchor with a 2-0 non-absorbable braided suture (Micro Corkscrew FT with 2-0 FiberWire, Arthrex, Naples, FL, USA) was placed along the caudal border of the lateral femoral condyle and the suture was placed hemi-circumferentially around the patella, passing through the proximal patellar tendon, lateral retinaculum, and distal patellar tendon, and securely tied.

The allogenic calcaneal tendon was trimmed to the appropriate length (4 cm) to cover the patellar ligament and quadriceps tendon. With the stifle positioned in full extension, components of the grafted tendon were individually sutured to the host tissue while maintaining proximal traction on the graft. The allogenic superficial digital flexor tendon was anchored to the patellar ligament and the central portion of the quadriceps tendon using a simple interrupted suture with a 3-0 PDS. The lateral (the conjoined tendon of the gracilis, semitendinosus, and biceps femoris muscles) and medial (the gastrocnemius tendon) flap of the allograft was sutured to the lateral and medial retinaculum, as well as the lateral and medial portions of the quadriceps tendon, respectively, using 3-0 PDS in a simple interrupted suture pattern (Figure 3H). The stifle was placed through a range of motion, and the patella tacked appropriately in the trochlear groove and did not luxate. A swab of the surgical site was obtained for bacterial culture prior to closure. The subcutaneous tissue and skin were closed in a routine manner.

## 3. Results

**Figure 4:**
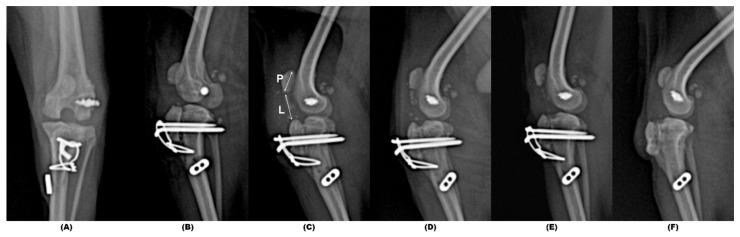
Immediate postoperative craniocaudal (**A**) and mediolateral (**B**) radiographic images, alongside sequential postoperative mediolateral radiographic images at immediate (**C**), 4 weeks (**D**), 12 weeks (**E**), and 28 weeks (**F**) post-surgery, with a PLL (marked as L in (**C**)): PL (marked as P in (**C**)) of 1.42, 1.46, 1.54 and 1.54, respectively. The patella remains normally positioned within the trochlear groove across all stages. The reconstruction of the tibial tuberosity using a calcaneus bone block allograft is distinctly visible. Due to skin irritation caused by the protruding ends of the Kirschner wires, pins and tension band wiring were removed at 26 weeks post-surgery. PLL, patellar ligament length; PL, patella length.

On postoperative radiographs, the patella was positioned appropriately in the trochlear groove. The allogenic bone block appeared to be securely attached to the tibia by the Kirschner wires and figure-of-eight wire (Figure 4). The postoperative patellar ligament length (PLL) to the patella length (PL) ratio in the left stifle was calculated to be 1.42, which was similar to a 1.41 PLL:PL ratio calculated on a radiograph of the contralateral stifle which was obtained postoperatively [19]. Following surgery, the dog was administered cephalexin (22 mg/kg PO, two times daily) for 1 week and carprofen (2.2 mg/kg PO, two times daily) for 2 weeks. The limb was immobilized in a modified Robert Jones bandage, and the dog was confined to a cage for 2 weeks. 

The dog was re-evaluated 2 weeks following surgery, and the incision had healed without complications. The dog was able to bear weight on the involved limb while standing and had a mild weight-bearing lameness with a decreased range of motion in the left stifle. Cultures of the allograft tendon and the surgical site obtained prior to closure had not yielded bacterial growth. Cage confinement was continued for an additional 2 weeks without bandaging the limb. Two weeks postoperatively, by 4 weeks postoperatively, the dog still had a mild weight-bearing lameness, and the owners were allowed to institute slow walks on a leash for 5–10 min twice daily; the duration of these walks was gradually increased to 30 min over 12 weeks. Both passive range of motion exercises and thermotherapy were also initiated twice daily at 4 weeks and continued until 12 weeks. The dog was re-evaluated 12 weeks following surgery. The dog had a weight-bearing lameness but could freely extend the right stifle with a near-normal range of motion in the joint. Radiographs obtained at 12 weeks revealed that interface between the grafted bone and the host bone could no longer be defined, and the patella continued to be properly positioned in the trochlear groove. Ultrasonography was performed 6 months postoperatively which revealed homogeneous continuity of the reconstructed patellar ligament between the grafted bone block and the distal pole of the patella. The Kirschner and tension band wires were removed 6 months following surgery as the protruding ends of the Kirschner wires were causing irritation of the overlying skin. By 7 months postoperatively, the dog was perceived to have a normal gait, and the patella was stable during manipulation. The dog could jump on and off a couch, although the thigh girth of the left pelvic limb was approximately 7% less than that of the unaffected limb. The left PLL:PL ratio had increased slightly to 1.54. The owner reported via telephone that the dog effectively used the left pelvic limb without lameness until the dog died of degenerative mitral valve disease 29 months after the surgery.

## 4. Discussion

A composite calcaneal tendon–bone block allograft was utilized to effectively restore the quadriceps extensor mechanism in the dog in the current case report. Reattachment of the patella ligament to the tibia in the dog described in this case report was complicated by resorption of the fragmented tibial tuberosity and extensive parenchymal loss of the distal patella ligament. Simple tenorrhaphy was not a viable treatment option due to the loss of the distal portion of the patellar ligament and the absence of the tibial tuberosity [20,21]. Additionally, direct attachment of the distal patellar ligament to the tibial bone seemed impractical due to a larger anticipated gap resulting from chronic quadriceps tendon contraction and debridement of the distal patellar ligament stump. Even if ligament-to-bone fixation were feasible, the repair would need to be protected for an extended period of time, given that ligament-to-bone healing typically progresses slower than ligament-to-ligament healing [22]. Therefore, any tenorrhaphy repair technique employed would have been unlikely to withstand the increased tension during surgery and the postoperative convalescent period [22]. Given these challenges, we elected to employ a novel surgical approach to effectively restore the function of the quadriceps extensor mechanism.

In assessing surgical strategies to bridge the gap between the remaining proximal portion of the patella ligament and an insertion point on the proximal tibia, we contemplated the use of either an isolated autogenous or allogenic tendon graft. Given the degree of tension the quadriceps mechanism would exert on any repair technique, we had concerns that the sutures placed at the graft–patellar ligament interface might dehisce and even greater concerns for dehiscence where the graft would be secured to the proximal tibia. The tendon graft-to-bone healing process involves multiple stages, including the development of fibrovascular interface tissue, gradual mineralization, and the penetration of bone into the outer layer of the grafted tendon [23]. Consequently, the time required for tendon graft-to-bone healing is typically longer compared to the relatively straightforward process of tendon-bone graft-to-bone healing, which involves direct bone growth and remodeling [23]. In addition, a previous study demonstrated that the bone-to-bone interface was superior to the bone–tendon interface in terms of mechanical strength, as indicated by higher failure stress [24]. Another in vivo comparative study recommended reconstructing the bone–tendon insertion interface by repairing between homogenous tissues [25]. Furthermore, studies have demonstrated that the integration of grafted tendon into host bone is slower and more susceptible to fixation failure compared to the integration of the host–graft osseous interface when using a composite tendon–bone allograft [16,17]. Additionally, there were concerns regarding abnormal biomechanical stress being placed on the patellofemoral articulation if we secured the patellar ligament in a caudal location due to loss of this dog’s tibial tuberosity, which might also contribute to dehiscence or hinder an optimal return to function [26,27,28].

These factors steered us to consider using a composite tendon–bone graft in this dog. Experimental studies in rabbits and clinical applications in human patients have reported strong, long-term integration at the graft–host osseous bone interface following implantation of allogenic composite tendon–bone grafts [29,30]. We felt that a composite tendon–bone graft would afford a secure means of performing a tenorrhaphy, provide reliable fixation of the ligament to the tibia, and contribute to the reconstruction of the normal morphology of the tibial tuberosity. In this case, an allograft was selected instead of an autograft to circumvent issues harvesting a suitable tendon–bone graft, which is particularly problematic in a toy breed dog. The use of an allograft eliminated any risks associated with donor site morbidity and the potential for extended surgical time associated with harvesting the graft [10,29]. Despite numerous studies reporting successful outcomes with bone–tendon allografts [6,12,13], concerns remain regarding infection, immune-mediated rejection, and the potential for nonunion or delayed union of the grafted bone segment [31,32]. In this case, the recipient bone bed consisted predominantly of cortical bone, with little exposed cancellous bone, which prompted our use of rhBMP-2 with HA to promote osseous union. The use of rhBMP-2 and HA has been shown to increase vascularization and osseous integration of cortical bone allografts [33]. Fortunately, there were no overt clinical or radiographic abnormalities suggestive of infection or immune rejection in the dog reported here. Caution is, however, advised when employing such allografts, as asepsis needs to be immaculate to prevent infection and the graft must be processed appropriately to mitigate the host’s immune response [31,32]. 

The allogenic bone block was positioned and secured along the lateral margin of the debrided tibial bone bed to the alignment of the quadriceps mechanism. An encircling patellar suture was placed and secured to the caudolateral femoral condyle as a supplemental restraint to medial luxation [34,35]. Patella luxation was never observed or elicited following surgery. 

After tenorrhaphy, it is imperative to maintain apposition of the tendon ends, or the tendon-to-bone interface, without gap formation until the healing process is complete [36,37,38]. A gap exceeding 3 mm between the apposed surfaces has been shown to reduce tensile strength and lead to suboptimal functional outcomes [36]. Relying solely on suture techniques has been shown to be inadequate in preventing gap formation throughout the healing phase of the postoperative convalescent period [37,38]. Consequently, supplemental stabilization methods are often utilized to protect tenorrhaphies during the initial postoperative convalescent period [38]. Placement of a trans-articular external fixator has been advocated to immobilize the stifle and to mitigate tensile forces exerted by the quadriceps muscles following patella tendon repair [38,39]. A fixator was not utilized in the dog in this case report because the dog’s weight was <2 kg. Fixators provide greater stability and are easier to apply and manage in large dogs and the risk of fracture through one of the pin tracks is a valid concern in small toy breed dogs [40,41]. A robust suture was placed, which engaged the proximal pole of the patella and secured to the proximal tibia, to mitigate tension on the repair in this dog. A similar supplement suture technique was shown to enhance biomechanical properties following primary patella ligament repair in a human cadaveric study [42]. Additionally, individual components of the allograft tendon were draped over the patella ligament and quadriceps tendon and secured to those structures using on-lay suturing, rather than performing a simple end-to-end tenorrhaphy. This method of augmented allogenic tendon repair yields immediate enhancement in the mechanical properties of the repair, surpassing those of primary tenorrhaphy alone [43]. During the suturing of the grafted tendon to the host tendon, the allografted tendon was tensioned while the stifle was maintained in full extension. This technique is supported by the results reported in a retrospective study, which found that successful restoration of the knee extensor mechanism is more likely when the allograft is tensioned rather than loosely attached during suturing in human patients with chronic patellar rupture [13]. 

This technique may be applied to chronic damage of the tendon–bone interface, such as the triceps brachii tendon–olecranon tuber and Achilles tendon–calcaneal tuberosity, when the tendons are completely transected and shortened, or the bony prominences are reabsorbed, making reconstruction between homogenous tissues difficult. For broader application of this technique, further study regarding in vivo biomechanical and histological examination would be necessary. There are several limitations to this technique, including difficulties in acquiring and storing the allograft, size differences between the graft and the host bed, and potential host responses to the allograft. Additionally, this study has limitations, as it is a single case report without biomechanical testing prior to application.

## 5. Conclusions

The tendon–bone allograft was particularly useful in the dog reported here because of the dog’s abnormal tibial tuberosity morphology. This surgical technique would appear to be applicable in analogous cases involving irreparable tibial avulsion fractures, when there is extensive patellar tendon loss or a primary tenorrhaphy is unlikely to be successful; however, further validation of this surgical technique through cadaveric studies and additional clinical trials is essential. 

## Data Availability

The data presented in this study are available on justified request from the corresponding author.
